# Elevated BCRP/ABCG2 Expression Confers Acquired Resistance to Gefitinib in Wild-Type EGFR-Expressing Cells

**DOI:** 10.1371/journal.pone.0021428

**Published:** 2011-06-23

**Authors:** Yun-Ju Chen, Wei-Chien Huang, Ya-Ling Wei, Sheng-Chieh Hsu, Ping Yuan, Heather Y. Lin, Ignacio I. Wistuba, J. Jack Lee, Chia-Jui Yen, Wu-Chou Su, Kwang-Yu Chang, Wen-Chang Chang, Tse-Chuan Chou, Chao-Kai Chou, Chang-Hai Tsai, Mien-Chie Hung

**Affiliations:** 1 Center for Molecular Medicine, China Medical University Hospital, Taichung, Taiwan; 2 Department of Pediatrics, China Medical University Hospital, Taichung, Taiwan; 3 Graduate Institute of Cancer Biology, China Medical University, Taichung, Taiwan; 4 Ph.D. Program for Cancer Biology and Drug Discovery, China Medical University, Taichung, Taiwan; 5 Graduate Institute of Basic Medical Science, China Medical University, Taichung, Taiwan; 6 Department of Molecular and Cellular Oncology, The University of Texas MD Anderson Cancer Center, Houston, Texas, United States of America; 7 Department of Pathology, The University of Texas MD Anderson Cancer Center, Houston, Texas, United States of America; 8 Department of Biostatistics, The University of Texas MD Anderson Cancer Center, Houston, Texas, United States of America; 9 Department of Pharmacology, National Cheng-Kung University, Tainan, Taiwan; 10 Institute of Clinical Medicine, National Cheng-Kung University, Tainan, Taiwan; 11 Internal Medicine, National Cheng-Kung University, Tainan, Taiwan; 12 Department of Chemical Engineering, National Cheng-Kung University, Tainan, Taiwan; 13 Department of Biotechnology, Asia University, Taichung, Taiwan; 14 Department of Healthcare Administration, Asia University, Taichung, Taiwan; 15 National Institute of Cancer Research, National Health Research Institutes, Tainan, Taiwan; 16 Department of Chemical Engineering, Tatung University, Taipei, Taiwan; Univesity of Texas Southwestern Medical Center at Dallas, United States of America

## Abstract

**Background:**

The sensitivity of non-small cell lung cancer (NSCLC) patients to EGFR tyrosine kinase inhibitors (TKIs) is strongly associated with activating EGFR mutations. Although not as sensitive as patients harboring these mutations, some patients with wild-type EGFR (wtEGFR) remain responsive to EGFR TKIs, suggesting that the existence of unexplored mechanisms renders most of wtEGFR-expressing cancer cells insensitive.

**Methodology/Principal Findings:**

Here, we show that acquired resistance of wtEGFR-expressing cancer cells to an EGFR TKI, gefitinib, is associated with elevated expression of breast cancer resistance protein (BCRP/ABCG2), which in turn leads to gefitinib efflux from cells. In addition, BCRP/ABCG2 expression correlates with poor response to gefitinib in both cancer cell lines and lung cancer patients with wtEGFR. Co-treatment with BCRP/ABCG2 inhibitors enhanced the anti-tumor activity of gefitinib.

**Conclusions/Significance:**

Thus, BCRP/ABCG2 expression may be a predictor for poor efficacy of gefitinib treatment, and targeting BCRP/ABCG2 may broaden the use of gefitinib in patients with wtEGFR.

## Introduction

The oncogenic EGFR tyrosine kinase, commonly overexpressed in a variety of solid tumors, plays important roles in cancer aetiology and progression, and thus is a rational target for cancer therapies. Selective small molecular inhibitors of EGFR tyrosine kinase (EGFR TKIs) have shown promising clinical activity in the last decade. Moreover, clinical studies reported that treatment of selective EGFR TKIs as monotherapy, including gefitinib (ZD1839, Iressa) and erlotinib (OSI-774, Tarceva), leads to tumor regression in 12–27% of advanced NSCLC patients [1], [2], [3].

Encouraging response to gefitinib is frequently observed in East Asian, female, adenocarcinoma histology, and non-smoking patients, and is closely associated with specific activating mutations in EGFR tyrosine kinase domain [4], [5], [6]. Since only a small population of unselected NSCLC patients has these mutations (about 10–15%), the clinical use of gefitinib is somewhat limited [4], [5], [6]. Nevertheless, 20–30% of NSCLC patients with amplified wild-type EGFR (wtEGFR) still demonstrated significant survival benefits from gefitinib and erlotinib treatment even though they showed lower response rate compared with patients with EGFR mutations [7], [8], [9]. Moreover, approximately 10–20% of gefitinib-responders were also found to have no identifiable EGFR mutations [6], [7], [8], [10], [11], [12], [13], suggesting that other unknown mechanisms may also contribute to the resistance to TKI treatment for most of patients with amplified wtEGFR. Therefore, the sensitivity to EGFR TKIs may not be determined only by these EGFR activating mutations.

To broaden the clinical use of EGFR TKIs, it is critical and timely to identify the determinants which render majority of wtEGFR-expressing cancer cells resistant to these drugs. Notably, a case report showed that a non-smoking female NSCLC patient with wtEGFR expression was initially responsive to gefitinib but ultimately developed acquired resistance without any detectable EGFR mutation. Interestingly, the expression of breast cancer resistance protein (BCRP/ABCG2), a well-known transporter of ATP-binding cassette (ABC) family involved in chemo-resistance [14], [15], was detected in the recurrent tumor from this patient [16]. Studies have shown that gefitinib not only acts as an inhibitor but also as a substrate for BCRP/ABCG2 [17], [18], [19], and enforced expression of BCRP/ABCG2 reduced the sensitivity of wtEGFR-expressing A431 cells to gefitinib [20]. Although these findings suggest a potential role of BCRP/ABCG2 in influencing the sensitivity to gefitinib, it remains unclear whether BCRP/ABCG2 expression is affected by gefitinib treatment and thus contributes to the resistance to this inhibitor.

In this study, acquisition of BCRP/ABCG2 expression was observed in wtEGFR-expressing and gefitinib-sensitive A431 cells after chronic treatment with gefitinib. Inhibition of BCRP/ABCG2 reduced gefitinib efflux and re-sensitized the cell line to this drug. The clinical correlation between BCRP/ABCG2 expression in tumor lesions and poor outcome was also observed in wtEGFR-expressing NSCLC patients who received gefitinib treatment. Our findings suggest that BCRP/ABCG2 expression may be a predictive factor for the sensitivity to gefitinib in patients with amplified wtEGFR and also a potential target for increasing the sensitivity to this inhibitor.

## Results

### BCRP/ABCG2 expression is elevated in acquired gefitinib-resistant A431/GR cells

**Figure 1:**
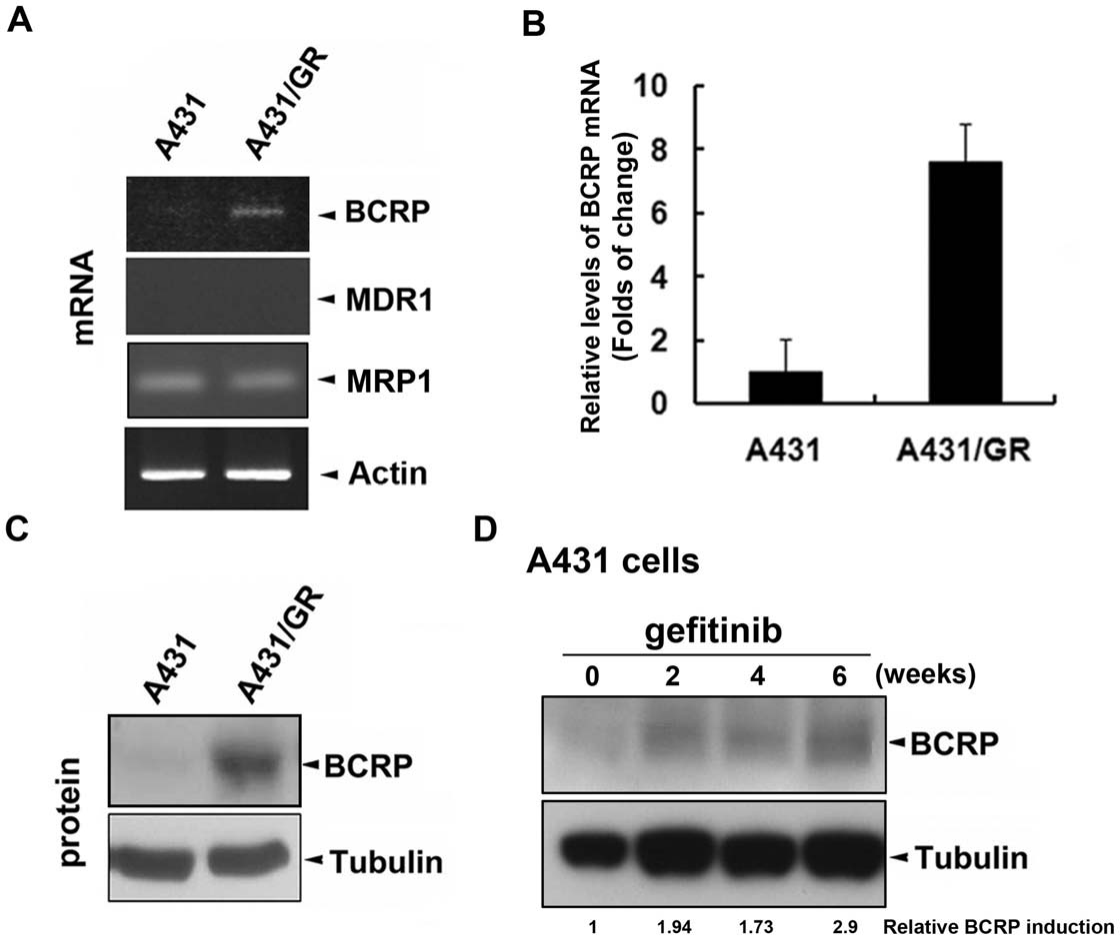
Acquired gefitinib-resistance in A431/GR cells induces BCRP/ABCG2 expression. *A–B*, The mRNA expression levels of BCRP/ABCG2, MDR1/ABCB1, and MRP1/ABCC1 in A431 and A431/GR cells were analyzed by RT-PCR (A) and the increased folds of BCRP/ABCG2 mRNA was measured by real-time PCR (B). Error bars in (B) denote s.e.m. (n = 3). Fold change of BCRP/ABCG2 mRNA expression was quantitated relative to the internal control, β-actin. *C*, The BCRP/ABCG2 protein expression level was analyzed by Western blot. *D*, A431 cells were treated with 1 µM gefitinib for the indicated time points, and cell extracts were analyzed by immunoblotting for BCRP/ABCG2 protein expression. The fold change of BCRP/ABCG2 protein expression was quantitated relative to the internal control, α-tubulin.

In this study, we employed wtEGFR-expressing and gefitinib-sensitive A431 epidermoid cell line and its gefitinib-resistant derivative, A431/GR [21] to address whether BCRP/ABCG2 plays a role in determining EGFR-TKI sensitivity in wtEGFR-expressing cancer cells. EGFR expression in the A431/GR cells retained the wild-type status as examined by cDNA sequencing (data not shown). In A431/GR cells, both mRNA (Figs. 1A and B) and protein (Fig. 1C) levels of BCRP/ABCG2 were significantly elevated as compared with that in parental A431 cells. However, the mRNA expression of multi-drug resistance 1 (MDR1)/ABCB1 and multi-drug resistance-related protein 1 (MRP1)/ABCC1, two other well-known ABC transporters related to chemo-resistance [14], [15], were not increased in response to gefitinib-resistance (Fig. 1A). In support of the results from A431/GR cells, the induction of BCRP/ABCG2 was also observed in parental A431 cells after treatment with gefitinib for 2 weeks, and continued for at least 6 weeks (Fig. 1D). Moreover, the elevation of BCRP/ABCG2 expression remained sustained even 7 days after gefitinib was removed from the culture medium of A431/GR cells (Fig. S1A). In parallel to this result, A431/GR cells cultured in gefitinib-free medium for 7 days still show the resistant phenotype as compared to those cultured in gefitinib-containing medium (Fig. S1B). These results suggest that the induction of BCRP/ABCG2 expression may not be reversible upon the withdrawal of gefitinib and reveal that BCRP/ABCG2 expression was specifically and irreversibly increased by gefitinib treatment, raising the possibility of the involvement of BCRP/ABCG2 in conferring acquired resistance to gefitinib.

### The gefitinib efflux in A431/GR cells is mediated by BCRP/ABCG2

**Figure 2:**
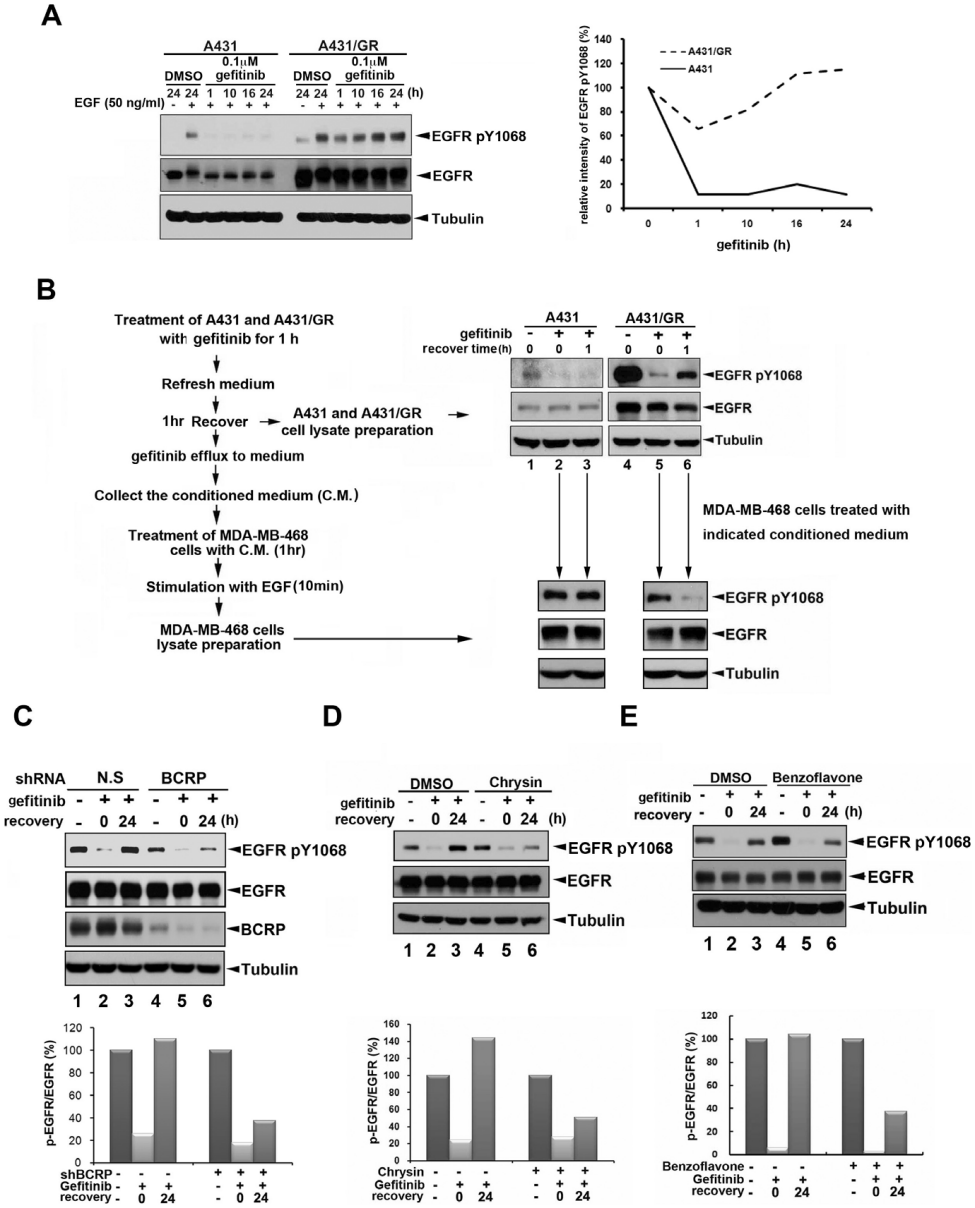
BCRP/ABCG2 shRNA and inhibitors reduce gefitinib efflux in A431/GR cells. *A*, A431 and A431/GR cells were cultured without gefitinib for 24 hr and then treated with DMSO or 0.1 µM gefitinib for the indicated period of time followed by 50 ng/ml EGF treatment for 10 minutes. Whole cell lysates were harvested and EGFR Tyr1068 phosphorylation was analyzed by Western blot (left) and quantitated (right). *B*, A431 and A431/GR cells were treated first with 5 µM gefitinib for 1 hr, and after incubation, the medium was removed and cells were replenished with fresh medium without the drug to allow recovery. After an hour incubation/recovery in the absence of gefitinib, the conditioned medium was collected and extracts were prepared to examine the recovery of EGFR activity by Western blot analysis (upper right). To test for presence of gefitinib efflux, MDA-MB-468 breast cancer cells were subjected to the conditioned medium (C.M.) collected from parental A431 and A431/GR cells after 1 hr incubation/recovery time. The effect of the conditioned medium on EGF-induced EGFR activity in MDA-MB-468 cells was further examined by Western blot (lower right). The detailed procedure is shown on the left. *C-E*, The recovery of EGFR activity from gefitinib inhibition in A431/GR cells infected with BCRP/ABCG2 shRNA virus (C) or treated with BCRP/ABCG2 inhibitors (D and E) was measured as described in Fig. 2B, left. Intensity was quantitated relative to the individual total EGFR expression (bottom).

Since gefitinib serves as both a substrate and an inhibitor for BCRP/ABCG2 [17], [18], [19], we further examined whether gefitinib is able to sustainably inhibit EGFR activity in A431/GR cells by detecting phosphorylation of EGFR Tyr1068 as an indicator. To this end, A431 and A431/GR cells were first cultured without gefitinib for 24 hrs and then treated with or without 0.1 µM gefitinib for indicated periods of time followed by EGF treatment for 10 minutes. As shown in Fig. 2A, gefitinib persistently inhibited the EGF-induced EGFR phosphorylation for at least 24 hrs in A431 cells. But the inhibitory effect of gefitinib on EGFR phosphorylation in A431/GR cells was partial and transient for up to 6 hrs, and this inhibitory effect was not observed if the pretreatment with gefitinib was over 10 hrs (Fig. 2A and Fig. S2). These observations imply that, in the presence of BCRP/ABCG2 expression, gefitinib transient inhibition of EGFR activity in A431/GR cells is probably due to a rapid efflux of this drug. In support of this notion, the transient inhibition of EGFR activity in A431/GR cells was prolonged when the concentration of gefitinib was increased (Fig. S2).

To further demonstrate that the transient EGFR inhibition by gefitinib in A431/GR cells was due to drug efflux, both A431 and A431/GR cells were treated first with gefitinib for 1 hr, and after incubation, the medium was removed and cells were replenished with fresh medium without the drug to allow recovery for another hour (as illustrated in Fig. 2B, left). After the 1 hr after incubation/recovery time, we collected the medium from parental A431 and A431/GR cells (hereafter referred to as the “conditioned medium”) and prepared cell extracts for Western blot analysis of EGFR activity. In A431/GR cells, EGFR Tyr1068 phosphorylation was recovered from the inhibition by gefitinib after the drug was removed and medium refreshed for 1 hr (Fig. 2B, top right, lanes 5 and 6) but not in the parental A431 cells (Fig. 2B, top right, lanes 2 and 3). We hypothesized that the reduction in the inhibition of EGFR Tyr1068 phosphorylation in A431/GR cells might be associated with gefitinib efflux, and therefore, the anti-EGFR tyrosine kinase activity of the conditioned medium from A431/GR cells would be higher than that of the parental A431 cells. To test this hypothesis, EGFR-overexpressing MDA-MB-468 breast cancer cells were treated with the conditioned medium collected as described above. We found that the conditioned medium from A431/GR cells significantly inhibited EGFR Tyr1068 phosphorylation in MDA-MB-468 cells (Fig. 2B, bottom right, lanes 5 and 6). In contrast, the conditioned medium from the parental A431 cells did not affect Tyr1068 phosphorylation of EGFR in MDA-MB-468 cells (Fig. 2B, bottom right, lanes 2 and 3). These results show that gefitinib is active in the A431/GR cells temporarily during the first 1-hr incubation but is then pumped out of the cell into the medium during the second 1-hr incubation with fresh medium, suggesting that gefitinib might be pumped out of the resistant cells much more easily than the sensitive cells.

Next, we examined whether blockage of BCRP/ABCG2 reduces the efflux of gefitinib in A431/GR cells. To this end, shRNA and inhibitors of BCRP/ABCG2 were used to block BCRP/ABCG2 function. As shown in Fig. 2C, inhibition of EGFR Tyr1068 phosphorylation by gefitinib was recovered within 24 hr in the control cells (Fig. 2C, lanes 1–3). However, silencing of BCRP/ABCG2 expression by shRNA reduced the recovery of EGFR Tyr1068 phosphorylation inhibited by gefitinib (Fig. 2C, lanes 4–6). Consistent with this finding, the inhibitory effect of gefitinib on EGFR activity in A431/GR cells was also enhanced in the presence of chrysin or benzoflavone (Figs. 2D and E), two well-established BCRP/ABCG2 inhibitors [22], [23], [24]. The percentage of EGFR Tyr1068 phosphorylation under BCRP/ABCG2 shRNA, chrysin, or benzoflavone treatment is shown (Figs. 2C–E; bottom panels). These results suggest that BCRP/ABCG2 expression is increased in the gefitinib-resistant cells, and thus facilitates the efflux of gefitinib.

### Blockage of BCRP/ABCG2 re-sensitizes A431/GR cells to gefitinib treatment

**Figure 3:**
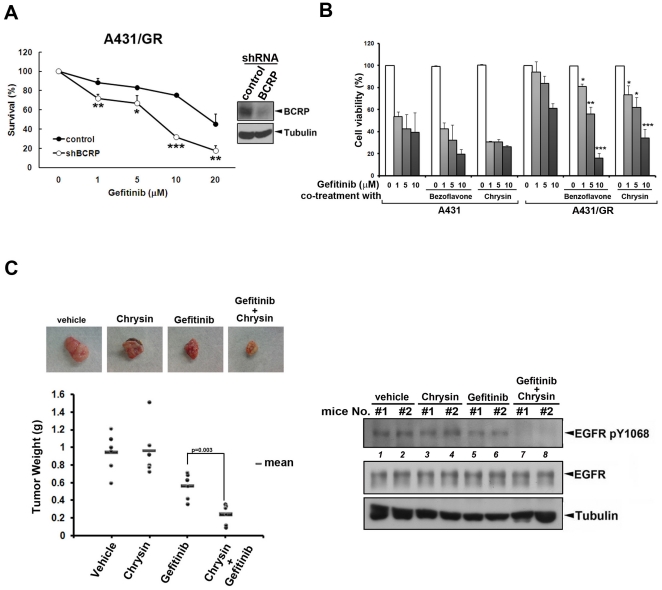
Inhibition of BCRP/ABCG2 overcomes the acquired resistance to gefitinib in A431/GR cells. *A–B*, The effect of BCRP/ABCG2 shRNA virus (A) or BCRP/ABCG2 inhibitors (B) on gefitinib sensitivity in A431/GR cells was measured by MTT assay. *C*, The effect of co-treatment of BCRP/ABCG2 inhibitor chrysin on the anti-tumor activity of gefitinib was examined in A431/GR-xenograft mouse model. After treatment of the indicated drug for 30 days, mice were sacrificed and tumors were weighed and measured (left). The xenograft tumor tissues from each group were homogenized and the total lysate were subjected to immunoblotting analysis with indicated antibodies (right). Error bars in A and B denote s.e.m. (n = 3). *, p<0.05; **, p<0.01; ***, p<0.001.

From the results above, inhibition of BCRP/ABCG2 activity may be able to reduce the acquired resistance to gefitinib by preventing the drug efflux. We further examined the cytostatic effect of gefitinib in A431/GR cells in the presence of BCRP/ABCG2 shRNA or BCRP/ABCG2 inhibitors. As expected, both silencing BCRP/ABCG2 (Fig. 3A) and treatment of chrysin or benzoflavone (Fig. 3B) significantly enhanced gefitinib-mediated cytostatic effect in A431/GR cells. However, these effects were not as obvious in A431 parental cells. Finally, a combined treatment with chrysin also enhanced gefitinib-mediated tumor regression in the A431/GR xenograft mouse model (Fig. 3C, left). EGFR activity was indeed reduced in the A431/GR-xenograft tumors treated with both chrysin and gefitinib but not in those treated with gefitinib or chrysin alone (Fig. 3C, right), supporting that co-targeting BCRP/ABCG2 may circumvent acquired gefitinib resistance both *in vitro* and *in vivo*.

### BCRP/ABCG2 expression is involved in intrinsic resistance to gefitinib

**Figure 4:**
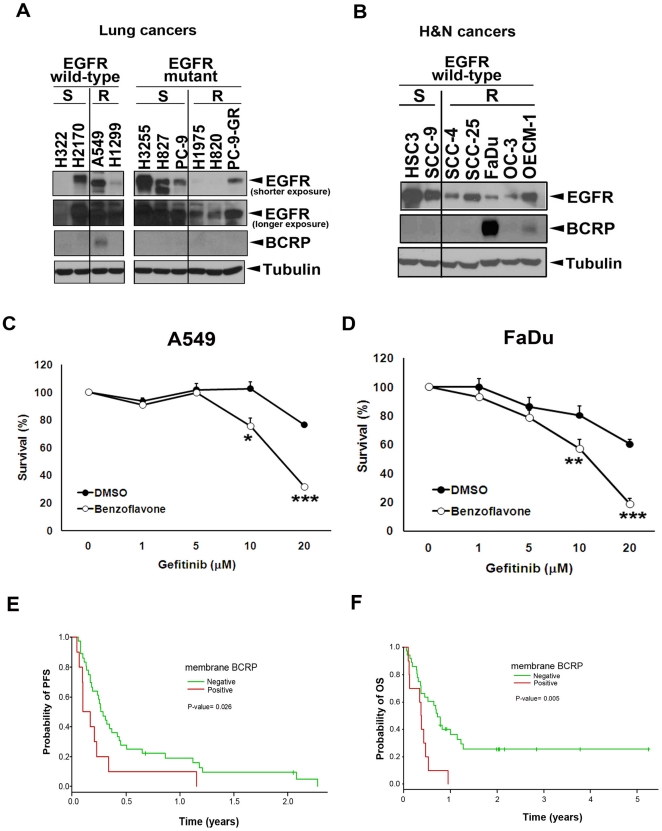
BCRP/ABCG2 expression is involved in intrinsic resistance to gefitinib. *A–B*, Western blot analysis of EGFR and BCRP/ABCG2 protein expression in lung (A) and head and neck (B) cancer cell lines bearing either wild-type or mutant EGFR. “S” and “R” represent gefitinib-sensitive and -resistant, respectively. *C–D*, The effect of gefitinib on cell viability of A549 (gefitinib-resistant; C) and FaDu (gefitinib-resistant; D) cells in the absence or presence of benzoflavone, a BCRP/ABCG2 inhibitor, was measured by MTT assay. Error bars in C and D denote s.e.m. (n = 3). *, p<0.05; **, p<0.01; ***, p<0.001. *E–F*, Progression-free survival (E) and overall survival (F) rates of membrane BCRP/ABCG2-positive or -negative lung cancer patients who received gefitinib therapy were analyzed as described in the “Materials and Methods”.

Next, to further strengthen the role of BCRP/ABCG2 in influencing gefitinib sensitivity, the correlation between BCRP/ABCG2 expression and gefitinib sensitivity was evaluated in various lung cancer cell lines, which express either wild-type or mutated EGFR [25], [26]. As shown in Fig. 4A, the BCRP/ABCG2 expression was only detected in the gefitinib-insensitive lung cancer cells bearing wtEGFR (A549). In contrast, neither gefitinib-sensitive nor gefitinib-resistant lung cancer cells carrying EGFR mutants showed BCRP/ABCG2 expression. In addition to lung cancer cells, head and neck cancer cells also frequently overexpress wtEGFR, but very few are sensitive to gefitinib. We found that two of five gefitinib-resistant head and neck cancer cell lines [27], including FaDu, and OECM-1 cell lines, express significant levels of BCRP/ABCG2 protein but was not detected in two gefitinib-sensitive HSC3 and SCC-9 cell lines (Fig. 4B). When A549 and FaDu cells were co-treated with BCRP/ABCG2 inhibitor benzoflavone, their sensitivity to gefitinib was significantly increased (Figs. 4C and D, respectively). These results imply that the intrinsic insensitivity of these cell lines to gefitinib might be, at least in part, due to the expression of BCRP/ABCG2.

To further validate the clinical relevance between BCRP/ABCG2 expression and intrinsic gefitinib resistance, lung tumor specimens from forty-nine patients (Table S1) were examined to identify the correlation between membrane BCRP/ABCG2 expression and the clinical benefit from gefitinib treatment. Although the association between membrane BCRP/ABCG2 expression and the best response to gefitinib did not reach statistical significance, the group with negative membrane BCRP/ABCG2 expression showed a higher percentage of stable disease and partial response (Table S2). Nevertheless, both progression free survival (PFS) and overall survival (OS) rates of these gefitinib-treated patients, as shown in Figs. 4E and F respectively, were significantly inversely associated with membrane BCRP/ABCG2 expression, indicating that patients with low membrane BCRP/ABCG2 expression may receive better survival benefit from gefitinib therapy. Together, our results suggest that membrane BCRP/ABCG2 expression may be another valuable marker to predict the clinical outcome of gefitinib-treated patients without EGFR activating mutations, and co-treatment with BCRP/ABCG2 inhibitors may increase the sensitivity to gefitinib and broaden its clinical use.

## Discussion

While the development of secondary EGFR mutations [5], [26] and alternative survival signals from other growth receptor activations such as c-Met [28], [29] have been widely known for conferring acquired gefitinib resistance of NSCLC patients who express activating EGFR mutations, very few related studies have reported the use of wtEGFR-expressing cells as the study model [21]. Here, we utilized a pair of epidermoid cancer cell lines expressing wtEGFR in an identical genetic background as a model to explore the determinants and the underlying mechanisms of acquired gefitinib resistance. Previously, it has been reported that BCRP/ABCG2 expression can be detected in a wtEGFR-expressing patient with acquired gefitinib resistance [16]. In the current study, we further validated this observation and showed that BCRP/ABCG2 expression, but not MDR1/ABCB1 and MRP1/ABCC1 expression, was indeed induced by chronic treatment of gefitinib in wtEGFR-expressing A431 cells (Fig. 1A) but not in mutEGFR-expressing PC-9 cells (Fig. 4A, PC-9 v.s. PC-9-GR). It was recently demonstrated that the BCRP/ABCG2 expression in the A431/GR cells is mediated by the Akt-dependent nuclear import of EGFR [30]. The induced BCRP/ABCG2 caused an efflux of gefitinib from the resistant but not sensitive A431 cancer cells. Therefore, co-targeting BCRP/ABCG2 can overcome the acquired gefitinib resistance both *in vitro* and *in vivo*. Although EGFR TKIs have been shown to serve as substrates of BCRP/ABCG2 [17], [18], [19], they have also been reported to be inhibitors of BCRP/ABCG2 [31], [32]. The molecular pathway that is described here provides a logical interpretation for the dual roles of gefitinib. The cross-resistance of A431/GR cells to erlotinib has previously been reported [21]. However, re-sensitization of A431/GR cells to erlotinib was not observed by using BCRP/ABCG2 inhibitor or shRNA (Figs S3A–C). Similar to our findings, co-treatment with other BCRP/ABCG2 inhibitors also had no effect on erlotinib activity in colon cancer cell lines, which might be due to the fact that erlotinib is not recognized as a typical substrate by BCRP/ABCG2 [31]. Therefore, BCRP/ABCG2 does not seem to be the major determinant of the cross-resistance to erlotinib in the cell model used in this study, and other mechanisms remain to be clarified. Since BCRP/ABCG2-positive tumors were found in 46% of advanced NSCLC patients [33], the BCRP/ABCG2-dependent drug resistance to gefitinib but not erlotinib might explain why erlotinib provides a better clinical outcome than gefitinib [7], [8] and may serve as a salvage treatment for NSCLC patients after failure of gefitinib treatment [34], [35]. In addition to BCRP/ABCG2, gefitinib and other TKIs have also been reported to function as inhibitors, but not substrates, of MDR1/ABCB1 [19], [32], [36], [37], another important ABC transporter for chemotherapeutic agents, indicating that MDR1/ABCB1 may be a potential target of TKIs to overcome chemoresistance rather than contributing to the drug resistance to these EGFR TKIs. However, we still cannot exclude the possibility that other members of ABC transporter family are involved in TKI resistance.

In addition to the gefitinib resistance, the increased expression of BCRP/ABCG2 also caused A431/GR cells to become cross-resistant to the chemotherapeutic agent doxorubicin, a well-established typical BCRP/ABCG2 substrate (Fig. S4A). The cross-resistance of A431/GR cells to doxorubicin can be reversed by benzoflavone (Fig. S4B). This finding suggests that BCRP/ABCG2-mediated drug efflux may be a common mechanism in gefitinib resistance and chemo-resistance, and raises an important issue of the timing in the use of gefitinib, a second-line therapeutic option originally approved by the U.S. FDA for advanced NSCLC patients who have failed systemic chemotherapy. Although a close association between prior chemotherapy and membrane BCRP/ABCG2 was not obtained in our current data due to the limited number of patients for the analysis of the impact of various types of chemotherapy on BCRP/ABCG2 induction (Table S3), expression of BCRP/ABCG2 has been found in several chemotherapy-resistant tumors [38], [39] and is correlated with the poor clinical outcome to platinum-based chemotherapy [33]. The BCRP/ABCG2-mediated gefitinib efflux may account for the poor clinical outcomes in most of the chemo-resistant patients while using gefitinib as second- or third-line therapy since results from several clinical trials revealed that the gefitinib response rate is higher in chemonaive than in chemotherapy-treated patients [40], [41], [42]. Our data also suggest that the membrane BCRP/ABCG2-negative patients have better survival benefits (Figs. 4E and F) and a higher response rate trend (Table S2) from gefitinib treatment than membrane BCRP/ABCG2-positive patients.

As the field of medicine moves toward an era of personalization, treatment decisions require the inputs of tumor-specific information. Our findings suggest that, in addition to the EGFR mutations, the status of BCRP/ABCG2 may also impact the effectiveness of gefitinib. Using BCRP/ABCG2 as another predictor of the clinical response to gefitinib will help us to decide on the use and priority of anti-cancer therapies. Our results also indicate that co-targeting BCRP/ABCG2 may not only overcome gefitinib resistance but also broaden the clinical use of gefitinib for various cancers with wtEGFR. Since intrinsic resistance was also observed in BCRP/ABCG2-negative cancer cells (Figs. 4A and B), the BCRP/ABCG2-mediated drug efflux may not be the only mechanism contributing to insensitivity of wtEGFR-expressing cancer cells to gefitinib, and other mechanisms await to be explored.

## Materials and Methods

### Cell lines and reagents

A431 and A431/GR cell lines were gifts from Dr. Carlos L. Arteaga (Vanderbilt-Ingram Cancer Center, Nashville, TN). Acquired gefitinib resistant cancer cells (A431/GR) were cultured in the presence of 1 µM gefitinib as described previously [21]. Commercially available gefitinib and erlotinib were purchased from the pharmacy of The University of Texas MD Anderson Cancer Center for both *in vitro* and *in vivo* experiments described in this study. Epidermal growth factor (EGF), chrysin, and benzoflavone were purchased from Sigma-Aldrich (St. Louis, MO). Anti-EGFR (SC-03) antibody from Santa Cruz Biotechnology, Inc. (Santa Cruz, CA) was used for EGFR immunoblotting. To detect EGFR autophosphorylation, a site-specific antibody against phospho-Y1068 from Cell Signaling (Danvers, MA) was used. BCRP/ABCG2 protein level was detected by anti-BCRP/ABCG2 antibody from Santa Cruz (SC58222) and by immunohistochemistry using anti-BCRP/ABCG2 antibody (MAB4146) from Chemicon (Billerica, MA).

### shRNA infection

BCRP/ABCG2 shRNA clones were purchased from the National RNAi Core Facility at Academia Sinica (Taipei, Taiwan). BCRP/ABCG2 shRNA virus packaging was prepared according to the manufacturer's instruction, and the BCRP/ABCG2 shRNA virus was used to infect target cells. Briefly, cells (8×10^3^ cells per well) were seeded in 96-well plates, and 24 hr after seeding, cells were infected with BCRP/ABCG2 shRNA virus at MOI 150. The next day, cells were refreshed with complete medium and then subjected to further indicated experiments.

### Cell proliferation assay

*In vitro* cell proliferation was carried out using 3-(4,5-dimethylthiazol-2-yl)-2,5-diphenyltetrazolium bromide (MTT) colorimetric assay. Briefly, cells (5–8×10^3^ cells per well) were seeded in 96-well plates, and 24 hr after seeding, cells were subjected to pre-treatments as indicated, including shRNA virus infection or pre-treatment of BCRP/ABCG2 inhibitors. After treatment of gefitinib, erlotinib, or doxorubicin for 48 or 72 hr, relative cell amounts were determined by adding 1 mg/ml MTT to each well. After a 3-hr incubation, the medium was removed, and MTT was solubilized in 100 µl of dimethyl sulfoxide (DMSO). The absorbance was measured at 570 nm.

### Xenograft mouse model

All animal works were done in accordance with a protocol approved by the Institutional Animal Care and Use Committee of China Medical University and Hospital (No. 100-61-N). *In vivo* cell growth was analyzed in an orthotopic epidermoid cancer mouse model [21]. Briefly, A431/GR cells (5×10^6^ cells) were injected subcutaneously into nude mice, and the tumor volumes were measured weekly. Once the tumor size reached 40 mm^3^, mice were subjected to oral treatment with saline, gefitinib (20 mg/kg), chrysin (100 mg/kg), or gefitinib (20 mg/kg) plus chrysin (100 mg/kg) every day. One month later, all mice were sacrificed and tumor size was weighed. The tumor weight was analyzed by a two-sided t-test.

### Immunohistochemical staining (IHC) of human lung tumor tissues

IHC was performed using anti-BCRP/ABCG2 antibodies (MAB4146, Chemicon). Briefly, the biotin-conjugated secondary antibody was incubated to form avidin-biotin-peroxidase complex. The immunoreaction was visualized by using aminoethylcarbazole chromogen as substrate. Protein staining was evaluated on a dual semi-quantitative scale combining staining intensity and percentage of positive cells in the cancer fields. The IHC score >0 or  = 0 was defined respectively as positive or negative for membrane BCRP/ABCG2 expression. Two investigators, independently and in a blind fashion, analyzed the protein expression. Fisher's exact and Spearman rank correlation tests were used for statistical analysis; p<0.05 was considered statistically significant. Lung cancer tumor tissues were collected from patients who received surgery at The University of Texas MD Anderson Cancer Center (Houston, TX). In both cancerous and non-cancerous sections, the fresh frozen tissue (stored in liquid nitrogen) and tissue embedded in paraffin were used for histology. All patients have signed the informed consent according to the IRB-approved protocol.

### Statistical analysis

Fisher exact test was used to test differences of category variables. The distribution of overall survival (OS) and progression-free survival (PFS) were estimated by the Kaplan-Meier method [43]. Log-rank test was performed to test the difference in survival between groups. Regression analyses of survival data based on the Cox proportional hazards model were conducted on PFS defined from the time of the start of gefitinib treatment to the time of progression or to the time of last contact, and OS was defined from the time of the start of gefitinib to the time of death or to the time of last contact. SAS version 9.1 and S-Plus version 7.0 were used to carry out the computations for all analyses.

## Supporting Information

Figure S1 **BCRP/ABCG2 expression and gefitinib resistance in A431/GR cells were sustained upon gefitinib withdrawal.** *A*, A431/GR cells were cultured in 1 µM gefitinib-containing medium. After 24 hrs of subculture, gefitinib was removed followed by collection of whole cell lysates on indicated days and then subjected to immunoblotting analysis with anti-BCRP and anti-tubulin antibodies. *B*, A431/GR cells were maintained with complete medium in the absence or presence of 1 µM gefitinib for 7 days and then subcultured with gefitinib-free medium and seeded in 96-well plate for viability assay. After 24 hrs of subculture, culture medium was refreshed and added with different concentrations of gefitinib for another 3 days. The cytostatic effect of gefitinib was measured by MTT assay.
(DOC)

Figure S2 **Transient inhibitory effect of gefitinib was observed in A431/GR cells.** A431/GR cells were cultured without gefitinib for 24 hrs. A431/GR cells were treated with 0.1, 0.5, and 1 µM gefitinib as indicated periods of time followed by 50 ng/ml EGF treatment for 10 minutes. EGFR Tyr1068 phosphorylation in A431/GR cells was analyzed by Western blot (top) and quantitated (bottom).
(DOC)

Figure S3 **BCRP/ABCG2 inhibition did not restore the cytostatic effect of erlotinib in A431/GR cells.** *A–B*, Effects of benzoflavone (A) and BCRP/ABCG2 shRNA (B) on erlotinib cytostatic activity in A431/GR cells were examined by MTT assay. *C*, Effect of BCRP/ABCG2 shRNA on the recovery of EGFR activity from erlotinib inhibition was examined as described in Fig. 2B. Error bars in A and B denote s.e.m. (n = 3).
(DOC)

Figure S4 **A431/GR cells were cross-resistant to doxorubicin due to the expression of BCRP/ABCG2.** *A*, Sensitivity of A431 and A431/GR cells to doxorubicin was measured by MTT assay. *B*, The effect of benzoflavone on doxorubicin cytotoxic activity in A431/GR cells was examined by MTT assay. Error bars in A and B denote s.e.m. (n = 3). *, p<0.05; **, p<0.01.
(DOC)

Table S1 **Selected patient characteristics.**
(DOC)

Table S2 **Association between membrane BCRP expression and best response to gefitinib.**
(DOC)

Table S3 **Association between membrane BCRP expression and patient's demographic/clinical characteristics.**
(DOC)
