# Supplementary material for: Elevated BCRP/ABCG2 Expression Confers Acquired Resistance to Gefitinib in Wild-Type EGFR-Expressing Cells
Source: PLoS One. 2011 Jun 23;6(6):e21428. doi: 10.1371/journal.pone.0021428 (PMC3121773; doi:10.1371/journal.pone.0021428)
Supplement: Figure S1 — BCRP/ABCG2 expression and gefitinib resistance in A431/GR cells were sustained upon gefitinib withdrawal. A, A431/GR cells were cultured in 1 µM gefitinib-containing medium. After 24 hrs of subculture, gefitinib was removed followed by collection of whole cell lysates on indicated days and then subjected to immunoblotting analysis with anti-BCRP and anti-tubulin antibodies. B, A431/GR cells were maintained with complete medium in the absence or presence of 1 µM gefitinib for 7 days and then subcultured with gefitinib-free medium and seeded in 96-well plate for viability assay. After 24 hrs of subculture, culture medium was refreshed and added with different concentrations of gefitinib for another 3 days. The cytostatic effect of gefitinib was measured by MTT assay. (DOC) [file pone.0021428.s001.doc]

**Supporting Information**

**
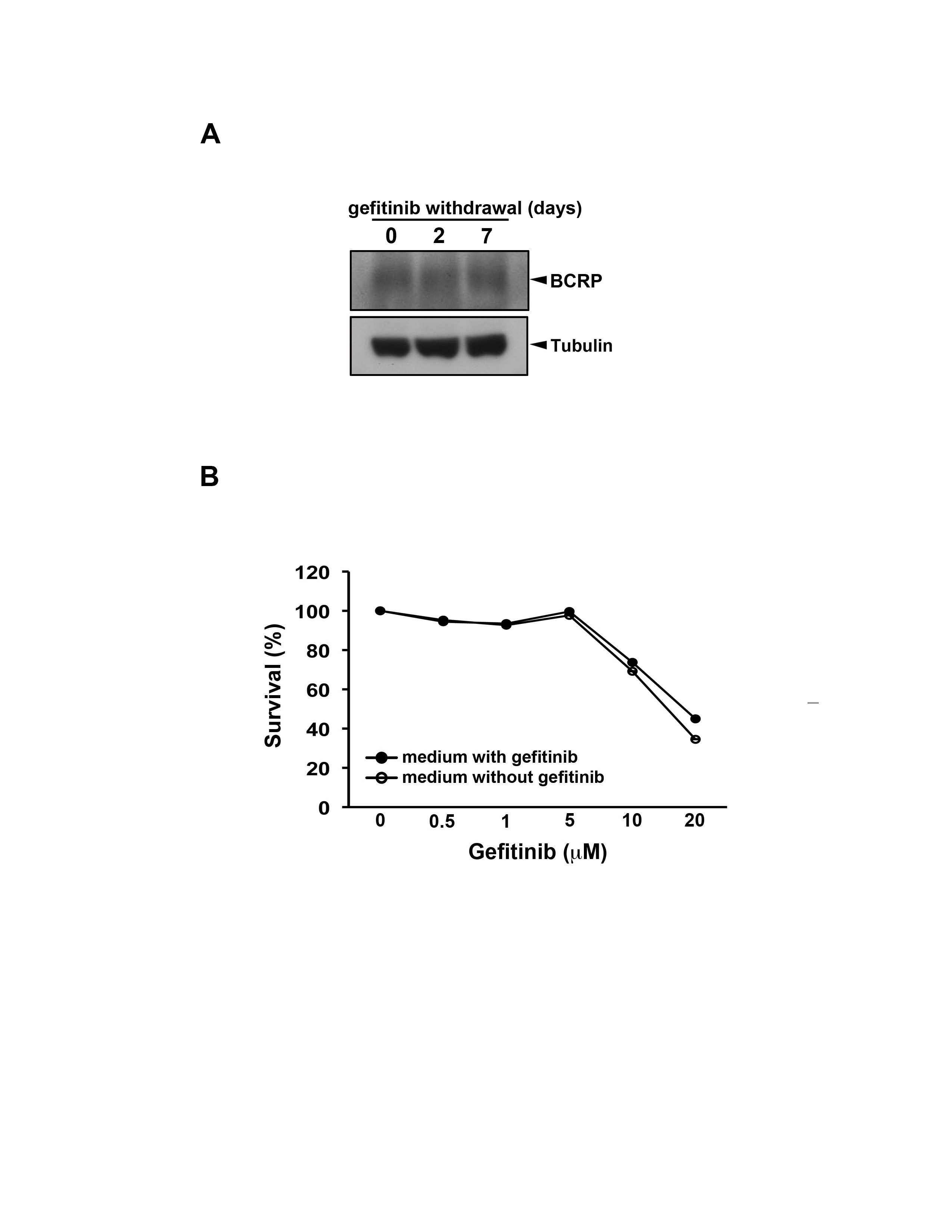
**

**Figure S1. BCRP/ABCG2 expression and gefitinib resistance in A431/GR cells were sustained upon gefitinib withdrawal.** *A,* A431/GR cells were cultured in 1 μM gefitinib-containing medium. After 24 hrs of subculture, gefitinib was removed followed by collection of whole cell lysates on indicated days and then subjected to immunoblotting analysis with anti-BCRP and anti-tubulin antibodies. *B*, A431/GR cells were maintained with complete medium in the absence or presence of 1 μM gefitinib for 7 days and then subcultured with gefitinib-free medium and seeded in 96-well plate for viability assay. After 24 hrs of subculture, culture medium was refreshed and added with different concentrations of gefitinib for another 3 days. The cytostatic effect of gefitinib was measured by MTT assay.
