# Supplementary material for: Elevated BCRP/ABCG2 Expression Confers Acquired Resistance to Gefitinib in Wild-Type EGFR-Expressing Cells
Source: PLoS One. 2011 Jun 23;6(6):e21428. doi: 10.1371/journal.pone.0021428 (PMC3121773; doi:10.1371/journal.pone.0021428)
Supplement: Figure S2 — Transient inhibitory effect of gefitinib was observed in A431/GR cells. A431/GR cells were cultured without gefitinib for 24 hrs. A431/GR cells were treated with 0.1, 0.5, and 1 µM gefitinib as indicated periods of time followed by 50 ng/ml EGF treatment for 10 minutes. EGFR Tyr1068 phosphorylation in A431/GR cells was analyzed by Western blot (top) and quantitated (bottom). (DOC) [file pone.0021428.s002.doc]

**Supporting Information**

**
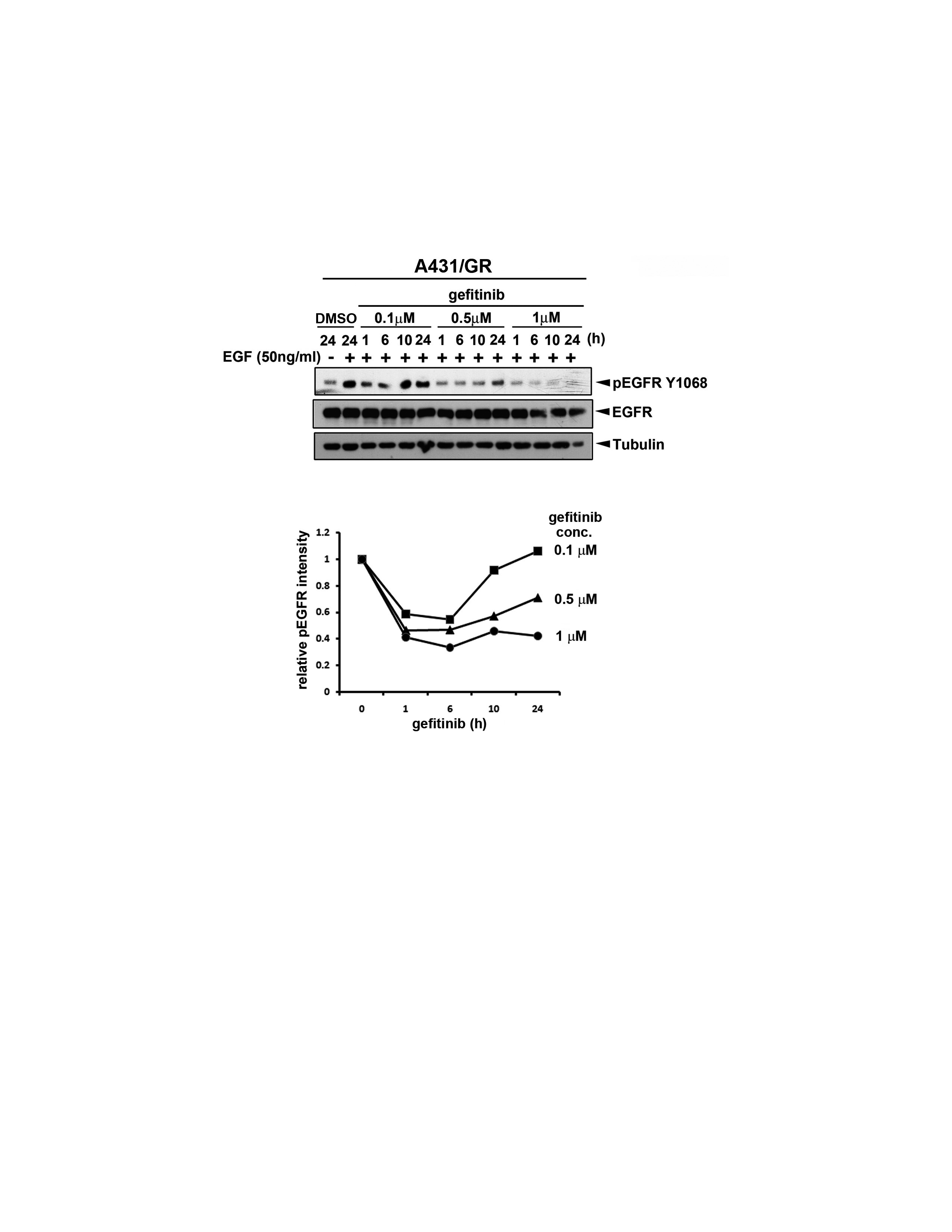
**

**Figure S2. Transient inhibitory effect of gefitinib was observed in A431/GR cells.** A431/GR cells were cultured without gefitinib for 24 hrs. A431/GR cells were treated with 0.1, 0.5, and 1 μM gefitinib as indicated periods of time followed by 50 ng/ml EGF treatment for 10 minutes. EGFR Tyr1068 phosphorylation in A431/GR cells was analyzed by Western blot (top) and quantitated (bottom).
