# Supplementary material for: Elevated BCRP/ABCG2 Expression Confers Acquired Resistance to Gefitinib in Wild-Type EGFR-Expressing Cells
Source: PLoS One. 2011 Jun 23;6(6):e21428. doi: 10.1371/journal.pone.0021428 (PMC3121773; doi:10.1371/journal.pone.0021428)
Supplement: Figure S3 — BCRP/ABCG2 inhibition did not restore the cytostatic effect of erlotinib in A431/GR cells. A–B, Effects of benzoflavone (A) and BCRP/ABCG2 shRNA (B) on erlotinib cytostatic activity in A431/GR cells were examined by MTT assay. C, Effect of BCRP/ABCG2 shRNA on the recovery of EGFR activity from erlotinib inhibition was examined as described in Fig. 2B. Error bars in A and B denote s.e.m. (n = 3). (DOC) [file pone.0021428.s003.doc]

**Supporting Information**

**
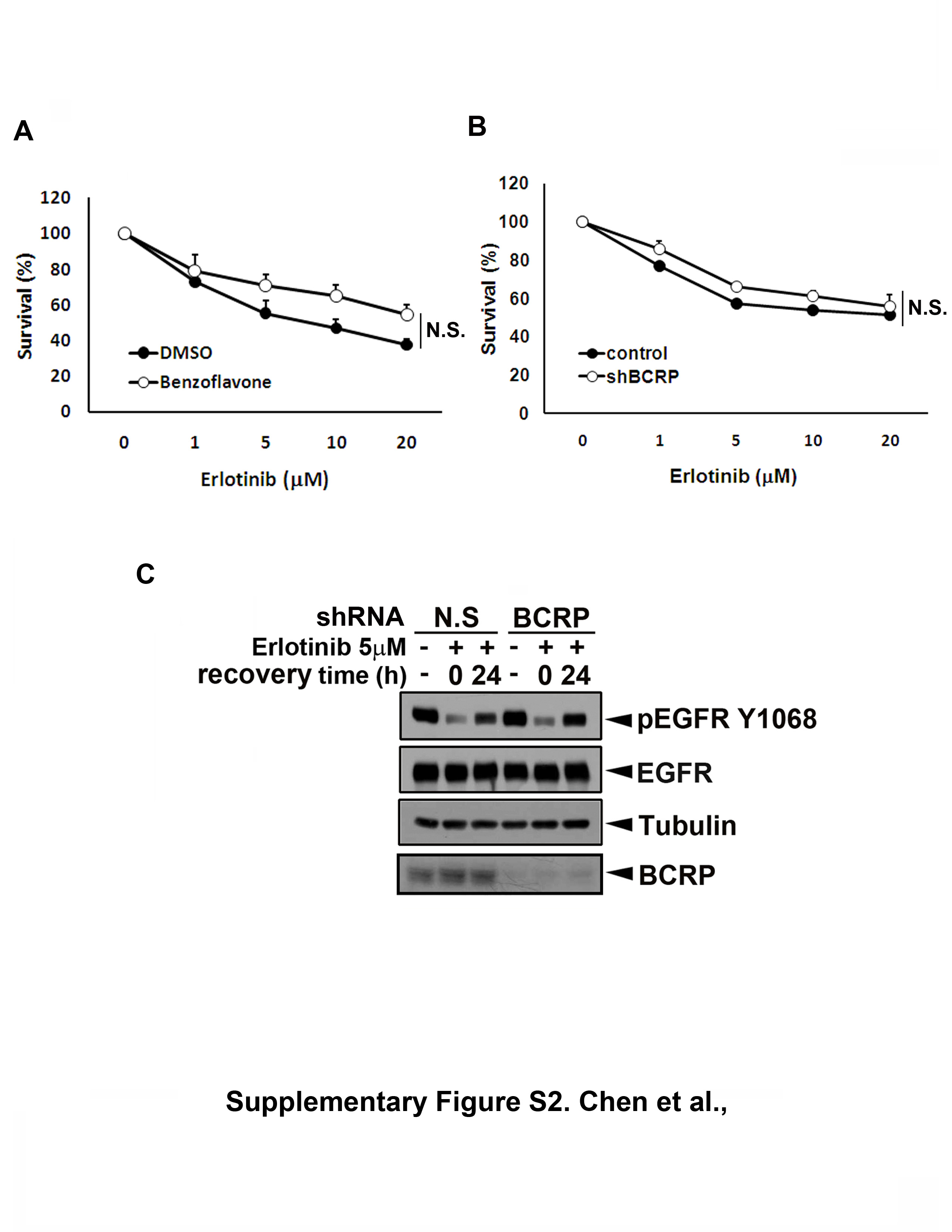
**

**Figure S3. BCRP/ABCG2 inhibition did not restore the cytostatic effect of erlotinib in A431/GR cells.** *A-B,* Effects of benzoflavone (A) and BCRP/ABCG2 shRNA (B) on erlotinib cytostatic activity in A431/GR cells were examined by MTT assay. *C,* Effect of BCRP/ABCG2 shRNA on the recovery of EGFR activity from erlotinib inhibition was examined as described in Fig. 2B. Error bars in A and B denote s.e.m. (n=3).
