# Supplementary material for: Elevated BCRP/ABCG2 Expression Confers Acquired Resistance to Gefitinib in Wild-Type EGFR-Expressing Cells
Source: PLoS One. 2011 Jun 23;6(6):e21428. doi: 10.1371/journal.pone.0021428 (PMC3121773; doi:10.1371/journal.pone.0021428)
Supplement: Figure S4 — A431/GR cells were cross-resistant to doxorubicin due to the expression of BCRP/ABCG2. A, Sensitivity of A431 and A431/GR cells to doxorubicin was measured by MTT assay. B, The effect of benzoflavone on doxorubicin cytotoxic activity in A431/GR cells was examined by MTT assay. Error bars in A and B denote s.e.m. (n = 3). *, p<0.05; **, p<0.01. (DOC) [file pone.0021428.s004.doc]

**Supporting Information**

**
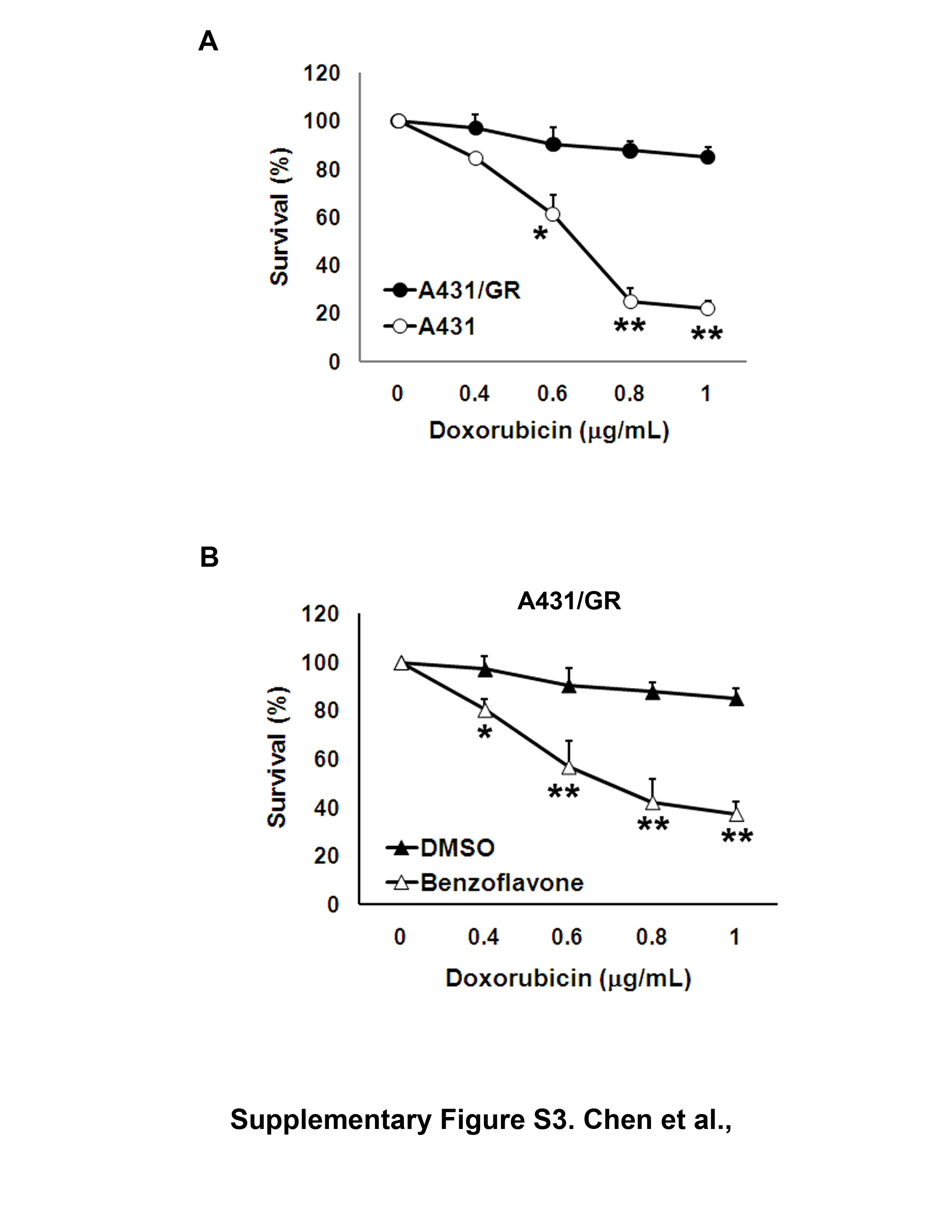
**

**Figure S4.** **A431/GR cells were cross-resistant to doxorubicin due to the expression of BCRP/ABCG2.** *A,* Sensitivity of A431 and A431/GR cells to doxorubicin was measured by MTT assay. *B,* The effect of benzoflavone on doxorubicin cytotoxic activity in A431/GR cells was examined by MTT assay. Error bars in A and B denote s.e.m. (n=3). *, p< 0.05; **, p<0.01.
