# Supplementary material for: Elevated BCRP/ABCG2 Expression Confers Acquired Resistance to Gefitinib in Wild-Type EGFR-Expressing Cells
Source: PLoS One. 2011 Jun 23;6(6):e21428. doi: 10.1371/journal.pone.0021428 (PMC3121773; doi:10.1371/journal.pone.0021428)
Supplement: Table S1 — Selected patient characteristics. (DOC) [file pone.0021428.s005.doc]

**Supporting Information**

**Table S1. Selected patient characteristics.**

| Table S1. Patient’s characteristics | | | |
| --- | --- | --- | --- |
| Variable | Category | Frequency Count | Percent of Total Frequency |
| Gender | Female | 26 | 53.06 |
|  | Male | 23 | 46.94 |
| Race | african american | 4 | 8.16 |
|  | Asian | 4 | 8.16 |
|  | caucasian | 38 | 77.55 |
|  | hispanic | 2 | 4.08 |
|  | missing | 1 | 2.04 |
| Smoking Status | Current | 17 | 34.69 |
|  | Former | 21 | 42.86 |
|  | Never | 11 | 22.45 |
| Performance Status | 0 | 1 | 2.04 |
|  | 1 | 29 | 59.18 |
|  | 2 | 12 | 24.49 |
|  | 3 | 7 | 14.29 |
| Histology | Adenocarcinoma | 27 | 55.10 |
|  | Large cell carcinoma | 1 | 2.04 |
|  | Non-small cell lung cancer | 15 | 30.61 |
|  | Squamous cell carcinoma | 6 | 12.24 |
| Best response to gefitinib treatment | Progress disease | 29 | 59.18 |
|  | Partial response | 2 | 4.08 |
|  | Stable disease 6 months | 3 | 6.12 |
|  | Stable disease<6 months | 9 | 18.37 |
|  | Stable disease>6 months | 6 | 12.24 |
| EGFR mutation | N/A | 2 | 4.08 |
|  | Non-detectable | 44 | 89.80 |
|  | Yes | 3 | 6.12 |
| EGFR mutation type | Non-detectable | 46 | 93.88 |
|  | E18 | 1 | 2.04 |
|  | E19 | 2 | 4.08 |
