# Supplementary material for: Elevated BCRP/ABCG2 Expression Confers Acquired Resistance to Gefitinib in Wild-Type EGFR-Expressing Cells
Source: PLoS One. 2011 Jun 23;6(6):e21428. doi: 10.1371/journal.pone.0021428 (PMC3121773; doi:10.1371/journal.pone.0021428)
Supplement: Table S2 — Association between membrane BCRP expression and best response to gefitinib. (DOC) [file pone.0021428.s006.doc]

**Supporting Information**

**Table S2. Association between membrane BCRP expression and best response to gefitinib.**

| Table S2. Association between membrane BCRP expression and best response to gefitinib | | | | | |
| --- | --- | --- | --- | --- | --- |
| Variable | Category (IHC score) | PD (%) | SD<=6mths | PR+SD>6mths | P-value |
| Membrane BCRP | Negative (=0) | 19(52.77) | 10(27.77) | 7(19.44) | 0.14347 |
|  | Positive (>0) | 9(90 ) | 1(10 ) | 0(0) |  |

PD: progression disease; SD: stable disease, PR: partial response;

* Fisher's Exact Test
