# Supplementary material for: Elevated BCRP/ABCG2 Expression Confers Acquired Resistance to Gefitinib in Wild-Type EGFR-Expressing Cells
Source: PLoS One. 2011 Jun 23;6(6):e21428. doi: 10.1371/journal.pone.0021428 (PMC3121773; doi:10.1371/journal.pone.0021428)
Supplement: Table S3 — Association between membrane BCRP expression and patient's demographic/clinical characteristics. (DOC) [file pone.0021428.s007.doc]

**Supporting Information**

Table S3. Association between membrane BCRP expression and patient’s demographic/clinical

characteristics.

| Table S3. Associations between membrane BCRP expression (Positive (>0) vs. Negative (=0))  and patient’s demographic/clinical characteristics | | | | |
| --- | --- | --- | --- | --- |
| Variable | Category | Positive membrane BCRP, N (%) | Negative membrane BCRP, N (%) | P-value* |
| Prior Chemotherapy regimen | No | 2(25 ) | 6(75 ) | 1.00000 |
|  | >=1 | 8(21.05) | 30(78.94) |  |

*** Fisher's Exact Test**
